# Combining talimogene laherparepvec with immunotherapies in melanoma and other solid tumors

**DOI:** 10.1007/s00262-017-1967-1

**Published:** 2017-02-25

**Authors:** Reinhard Dummer, Christoph Hoeller, Isabella Pezzani Gruter, Olivier Michielin

**Affiliations:** 1 Department of Dermatology, University of Zürich Hospital, Gloriastrasse 31, 8091 Zurich, Switzerland; 2 Medical University of Vienna, Vienna, Austria; 3 Amgen (Europe) GmBH, Zug, Switzerland; 4 Lausanne University Hospital, Lausanne, Switzerland

**Keywords:** Checkpoint inhibitors, Combination treatment, Immunotherapy, Melanoma, Solid tumors, Talimogene laherparepvec

## Abstract

Talimogene laherparepvec is a first-in-class intralesional oncolytic immunotherapy. In a recent Phase III trial (OPTiM), talimogene laherparepvec significantly improved durable response rate compared with subcutaneous granulocyte–macrophage colony-stimulating factor (GM-CSF). Overall response rate was also higher in the talimogene laherparepvec arm, and the greatest efficacy was demonstrated in patients with earlier-stage (IIIB, IIIC, or IVM1a) melanoma. Talimogene laherparepvec was well tolerated, with the majority (89%) of adverse events being grade 1 or 2. Preclinical studies have shown that talimogene laherparepvec exerts antitumor activity by selectively replicating within and destroying cancer cells, and through the release of tumor-associated antigens and expression of GM-CSF, which facilitates a wider antitumor immune response. It is hypothesized that combining talimogene laherparepvec with a systemic immunotherapy may, by bringing together complementary mechanisms of action, further enhance the efficacy of both agents. Indeed, talimogene laherparepvec is currently being assessed in combination with immune checkpoint inhibitors, including ipilimumab and pembrolizumab, in trials for melanoma and other solid tumors. Early results in melanoma indicate that the combination of talimogene laherparepvec with ipilimumab or pembrolizumab has greater efficacy than either therapy alone, without additional safety concerns above those expected for each monotherapy. In this review, we discuss the latest results from trials assessing talimogene laherparepvec in combination with other immunotherapies, provide an overview of ongoing and upcoming combination trials, and suggest future directions for talimogene laherparepvec in combination therapy for solid tumors.

## Introduction

Talimogene laherparepvec is a first-in-class intralesional oncolytic viral therapy that, based on data from the recent Phase III Oncovex (GM-CSF) Pivotal Trial in Melanoma (OPTiM) trial in stage IIIB–IVM1c melanoma, became the first oncolytic immunotherapy to be approved by the United States (US) Food and Drug Administration (FDA) [1]. In the wake of FDA approval and following a subgroup analysis of patients with stage IIIB–IVM1a melanoma from the Phase III study, talimogene laherparepvec also became the first oncolytic immunotherapy approach to be approved in Europe, where it is indicated for adults with unresectable stage IIIB, IIIC, or IVM1a melanoma with no bone, brain, lung, or other visceral disease [2, 3]. In Australia, the Therapeutic Goods Administration has approved talimogene laherparepvec as monotherapy for the treatment of melanoma in patients with unresectable cutaneous, subcutaneous, or nodal lesions after initial surgery [4].

### Mechanism of action

Talimogene laherparepvec was generated from herpes simplex virus type 1 (HSV-1), a contagious, lytic, and human pathogen around 200 nm in diameter with a large genome (152 kb) that was considered a suitable vector due to its well-characterized biology [5–7]. To initiate infection, HSV-1 attaches to cell surface receptors before rapid fusion of the viral envelope with the cell membrane occurs, enabling transport of the viral DNA to the cell nucleus [5]. Initial attachment is mediated by the interaction of viral glycoproteins with cell surface heparin sulfate [5]. This is followed by viral binding with cell surface receptors, such as nectin-1 and herpesvirus entry mediator A, which are broadly expressed across a wide variety of human cell types [5]. Talimogene laherparepvec is an attenuated form of HSV-1 that has been modified to diminish viral pathogenicity as well as to induce selective tumor lysis and increase antigen presentation [8]. Specifically, both copies of the gene encoding ICP34.5 have been deleted, which is expected to reduce pathogenicity and provide tumor selective replication due to the oncogenic disruption of the protein kinase R (PKR) pathway [8]. In place of *ICP34.5*, the gene encoding human regulatory cytokine granulocyte–macrophage colony-stimulating factor (GM-CSF) has been inserted [8]. GM-CSF enhances the immune response to tumors [8], attracts and induces myeloid precursor cells to proliferate and differentiate [9], and recruits and stimulates dendritic cells [10]. Talimogene laherparepvec is also modified by deletion of the *ICP47* gene, which prevents ICP47 from blocking antigen presentation, thereby helping to restore immunogenicity [8]. This deletion also leads to elevated expression of the HSV *US11* gene as an immediate early gene, rather than late gene, which enables US11 to block PKR activity before PKR is able to terminate protein synthesis, leading to increased replication of ICP34.5-deleted HSV-1 in tumor cells [8, 11].

Following administration of talimogene laherparepvec, selective intratumoral replication and subsequent oncolysis directly destroys cancer cells and releases progeny viruses, tumor-associated antigens and danger-associated molecular factors [12]. The progeny viruses then infect other local tumor cells, intensifying the ‘danger’ signals and propagating the antitumor effect [8, 12]. GM-CSF helps prime and induce tumor-specific immunity by promoting the maturation and function of dendritic cells, which may activate antitumor T cells through the presentation of the processed tumor-associated antigens. Activated T cells can then proliferate and migrate to distant tumor sites, where they may recognize tumor cells with matching antigen profiles. These properties differentiate talimogene laherparepvec from other intralesional agents, which are in earlier stages of development and are often replication deficient (Table 1).

**Table 1:** Other intralesional therapies in development or discontinued

Agent	Description and mode of action	Replication competent	Trial phase	Suitable for systemic delivery?
Allovectin-7 (velimogene aliplasmid) [13, 14]	A plasmid/lipid complex encoding HLA-B7 and ß2 microglobulin, both components of MHC-I	No	Discontinued	No
ALVAC GM-CSF [15]	Viral vector system using recombinant canarypox virus for local GM-CSF gene expression; GM-CSF activates dendritic cells, macrophages and granulocytes	No	I	No
ALVAC IL-2 [15]	Viral vector system using recombinant canarypox virus for local IL-2 gene expression. IL-2 stimulates T-cell proliferation, induces activation of cytotoxic T lymphocytes and natural killer cells	No	I	No
CVA21 (CAVATAK) [16]	An oncolytic and immunotherapeutic strain of Coxsackievirus A21 that leads to cell lysis and enhancement of antitumor immune responses	Yes	II	Yes
Pexastimogene devacirepvec (JX-594) [17]	Modified vaccinia virus with thymidine kinase deletion and GM-CSF insertion; stimulates antitumor immunity	Yes	I/II	Yes
PV-10 [18]	A water-soluble xanthene dye that, when given as an intralesional injection, leads to tumor ablation	No	III	No
TG1024 (adenovirus IL-2) [19]	Recombinant adenovirus construct, expressing genes for IL-2, which stimulates T-cell proliferation, induces activation of cytotoxic T lymphocytes and natural killer cells	No	I/II	No
Xenogenic plasmid IL-12 [20]	Plasmid DNA encoding IL-12, which enhances the immune capacity of natural killer cells and T cells	No	I/II	No

*HLA* human leukocyte antigen, *IL* interleukin, *MHC* major histocompatibility complex, *NA* not reported

### Preclinical and clinical experience

Preclinical models have demonstrated talimogene laherparepvec-induced tumor lysis and augmented antitumor immune responses in a number of different cancer cell lines and animal models [8, 21]. Data showing that HSV-1 antigen and DNA are selectively expressed in tumors injected with talimogene laherparepvec [22] which provides evidence that the direct antitumor effects of talimogene laherparepvec occur mainly at the injection site. In addition, the increased area occupied by CD8+ T cells within both injected and uninjected tumors show the development of an indirect systemic antitumor immune response following talimogene laherparepvec injection [23]. In murine models, both injected and uninjected tumors were reduced or cleared and mice also developed resistance to subsequent challenge with the same tumor cells [8, 21, 22]. Prolonged survival following treatment with talimogene laherparepvec was also seen in a mouse tumor model [22].

Clinical trials have demonstrated the safety and efficacy of talimogene laherparepvec in patients [6, 24, 25]. The first-in-human study was conducted in pre-treated patients with breast, head and neck, gastrointestinal cancers, and melanoma, to determine the safety profile and biological activity of talimogene laherparepvec and to identify a suitable dose schedule for future studies [24]. Talimogene laherparepvec was well tolerated with no maximum-tolerated dose reached (which enabled a multi-dosing schedule to be defined) and biological activity (virus replication, GM-CSF expression, local reactions, and HSV-1 antigen-associated tumor necrosis) was observed [24].

A Phase II trial evaluated the efficacy and safety of talimogene laherparepvec in patients with unresectable, stage IIIC-IV malignant melanoma (clinicaltrials.gov identifier: NCT00289016) [25]. Melanoma was selected for this study due to the availability of accessible lesions for direct injection and because an active role for the immune system has been implicated in this type of cancer. The Phase II trial reported a 26% overall response rate (ORR) in talimogene laherparepvec-treated patients and limited toxicity [25]. Early studies also identified the accumulation of MART-1-specific CD8+ T cells in both injected and uninjected lesions, suggesting both local and systemic immune activity [26].

**Fig. 1:**
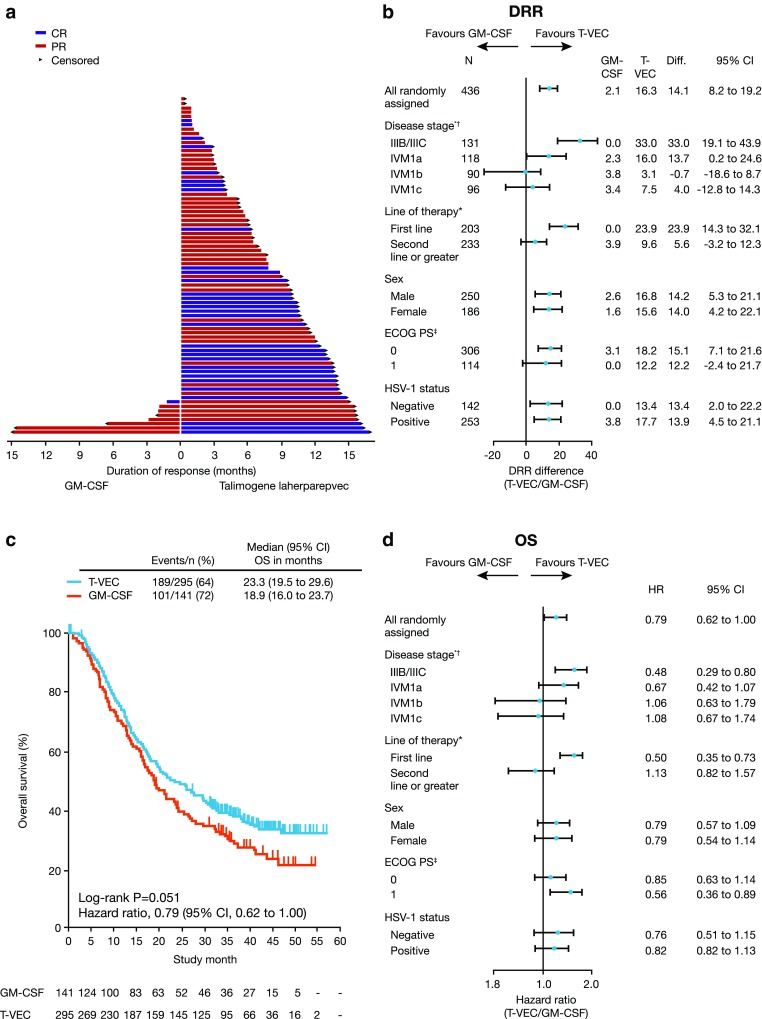
Key efficacy data from the Phase III talimogene laherparepvec OPTiM clinical trial [6]*. **a** Duration of response for all patients with response per endpoint-assessment committee (EAC) assessment. Duration of response was defined as longest period of response from entering response to first documented evidence of patient no longer meeting criteria for response. *Arrows* indicate patients for whom duration of response was censored at last tumor assessment, because there was no evidence (per EAC assessment) that their response had ended. **b** DRR in patient subgroups defined by key baseline characteristics. **c** Primary analysis of OS in intent-to-treat population. **d** OS in patient subgroups defined by key baseline characteristics. *Reprinted with permission from Andtbacka et al. [6]. © 2017 American Society of Clinical Oncology. All rights reserved. *CR* complete response, *DRR* durable response rate, *ECOG* Eastern Cooperative Oncology Group, *GM-CSF* granulocyte–macrophage colony-stimulating factor, *HR* hazard ratio, *HSV-1* herpes simplex virus-1, *OS* overall survival, *PS* performance status, *PR* partial response, *T-VEC* talimogene laherparepvec. *p < 0.001 per Gail and Simon [28] quantitative treatment by covariate interaction test (for DRR). ^†^One patient in the talimogene laherparepvec arm had unknown disease stage. ^‡^Twelve patients in the GM-CSF arm and four in the talimogene laherparepvec arm had unknown ECOG status

These positive results led to the prospective, randomized, open-label Phase III OPTiM trial (clinicaltrials.gov identifier: NCT00769704) [6]. The trial included 436 treatment-naïve and previously treated patients with unresectable stage IIIB–IVM1c melanoma from May 2009 until July 2011. Patients were randomized at a two-to-one ratio to treatment with intralesional talimogene laherparepvec (*n* = 295) or subcutaneous recombinant GM-CSF (*n* = 141) [6]. GM-CSF was considered to be a valid comparator at the time the study was designed/conducted, as available data suggested that it has some antitumor activity in melanoma and is associated with minimal toxicity [27]. The primary endpoint for OPTiM was durable response rate (DRR), defined as the rate of complete response (CR) plus partial response (PR) beginning within 1 year of treatment and maintained for ≥6 months continuously, as assessed by an endpoint-assessment committee [6]. In the overall population, talimogene laherparepvec significantly improved DRR compared with subcutaneous GM-CSF (16 vs 2%, respectively; *p* < 0.001), with efficacy most pronounced in patients with earlier-stage metastatic disease [6]. Specifically, the difference in DRR between talimogene laherparepvec and GM-CSF was greater in patients with stage IIIB/C (33 vs 0%) and IVM1a disease (16 vs 2%) compared with patients with stage IVM1b (3.1 vs 3.8%) and IVM1c (7.5 vs 3.4%) disease (Fig. 1) [6]. In the overall population, a median survival difference of 23 vs 19 months was observed with talimogene laherparepvec vs GM-CSF (hazard ratio [HR]: 0.79; 95% confidence interval [CI]: 0.62, 1.00; *p* = 0.051) (Fig. 1) [6]. Further analysis showed talimogene laherparepvec’s effect on overall survival (OS) to be greater in patients with earlier-stage disease and in those with treatment-naïve disease (Fig. 1) [6]. In patients with stage IIIB–IVM1a melanoma, median OS was longer in the talimogene laherparepvec arm (41 months; 95% CI, 31 months, not evaluable), compared with the GM-CSF arm (21.5 months; 95% CI, 17, 30 months). Talimogene laherparepvec was well tolerated in OPTiM; the most common adverse events (AEs) occurring in patients receiving talimogene laherparepvec were fatigue, chills, and pyrexia [6]. The majority (89%) of AEs were grade 1 or 2. Cellulitis was the only grade 3 or 4 AE to occur in ≥2% of talimogene laherparepvec-treated patients, and no fatal treatment-related AEs occurred during the study [6].

There is room to increase these observed beneficial effects with talimogene laherparepvec. A treatment approach that shows promise is the combination of different immunotherapies, which has the potential to improve efficacy relative to either therapy alone [29]. In this review, we discuss the potential for talimogene laherparepvec to be used in combination with other immunotherapies and revisit the ongoing and upcoming talimogene laherparepvec combination trials.

## Rationale for combining talimogene laherparepvec with other immunotherapies

**Fig. 2:**
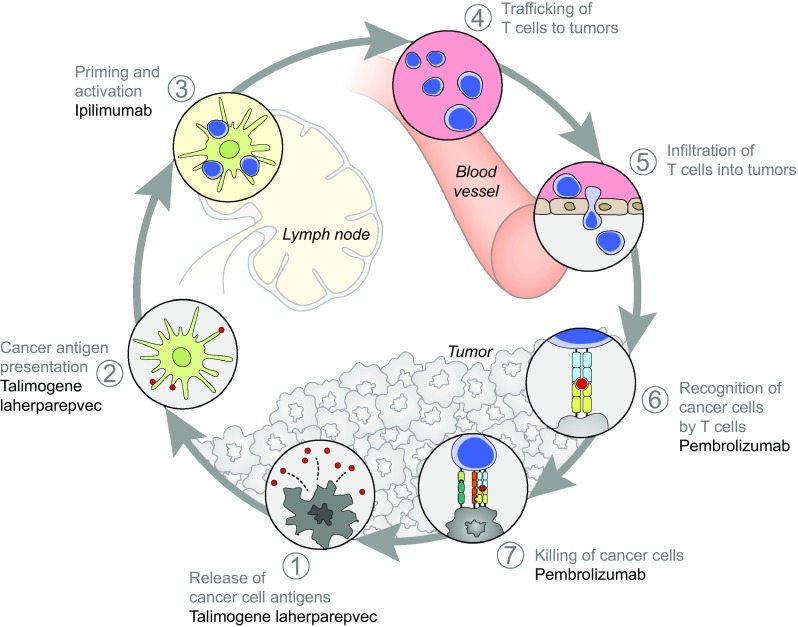
Mechanism of action of talimogene laherparepvec combined with ipilimumab or pembrolizumab [36]^*^. Talimogene laherparepvec would act to enhance the cancer–immunity cycle through inducing the death of tumor cells causing the release of TDAs. Talimogene laherparepvec would also enhance the activation and recruitment of dendritic cells through the production of GM-CSF, thereby causing increased processing of TDAs by the dendritic cells. Ipilimumab could enhance the action of talimogene laherparepvec to further boost the cancer–immunity cycle by enhancing the priming and activation of T cells by dendritic cells presenting TDAs. Pembrolizumab could enhance the action of talimogene laherparepvec to further boost the cancer–immunity cycle by enhancing the recognition and killing of tumor cells by T cells. ^*^Reprinted from Immunity, Volume 39, Chen and Mellman [36], Page 7, Copyright (2016), with permission from Elsevier. *GM-CSF* granulocyte–macrophage colony-stimulating factor, *TDA* tumor-derived antigen

Talimogene laherparepvec has considerable local immune activity, with intralesional administration resulting in responses (≥50% regression) in 64% of injected lesions during OPTiM [30]. A 50% reduction in tumor size was also seen in 34% of non-injected non-visceral lesions and in 15% of visceral lesions, indicating that talimogene laherparepvec also induces systemic antitumor immunity and response [30]. While activity was observed at distant metastases, it has been hypothesized that combining talimogene laherparepvec with other systemic immunotherapies may further enhance the efficacy of both agents. In this regard, talimogene laherparepvec’s potentially complementary mechanism of action with other approved immunotherapies supports its use in combination clinical trials (Fig. 2). The oncolytic properties of talimogene laherparepvec result in the release of tumor-derived antigens in an immune stimulatory microenvironment, local production of GM-CSF, and cross-priming of CD8+ T-cell responses by dendritic cells, which facilitate an immune response against the tumor [12]. However, immune responses can be evaded through the expression of immunosuppressive checkpoint receptors on the surface of T cells, such as CTL antigen-4 (CTLA-4) and programmed cell death 1 (PD-1). Checkpoint inhibitors, including CTLA-4 inhibitors (e.g., ipilimumab) and PD-1 receptor/ligand inhibitors (e.g., pembrolizumab, nivolumab, and atezolizumab), have established efficacy, acting systemically to enhance T-cell recruitment and prevent exhaustion of activated T cells [31–36]. CTLA-4 and PD-1/PD-L1 blockade can also reduce T-regulatory cell function, which may contribute to an antitumor response [35]. Combining talimogene laherparepvec with immune checkpoint inhibitors, therefore, has potential to augment tumor-specific immune responses and enhance the antitumor activity compared with either treatment alone. In this respect, a preclinical study demonstrated that an injected oncolytic immunotherapy combined with CTLA-4 blockade had enhanced activity in local and distant tumors compared with either agent alone [37].

The concept of combining immunotherapies with differing mechanisms of action was recently demonstrated in a randomized, double-blind, Phase III study assessing ipilimumab in combination with nivolumab in melanoma [29]. The median progression-free survival (PFS) was 11.5 months with combination therapy, versus 2.9 months with ipilimumab alone and 6.9 months with nivolumab alone [29]. However, significantly more treatment-related grade 3 or 4 AEs, including immune-related AEs, were seen with ipilimumab plus nivolumab (55%) compared with ipilimumab monotherapy (27%) or nivolumab monotherapy (16%) [29]. Non-overlapping toxicity profiles are important for patients to successfully tolerate treatment combinations. Therefore, the relatively low rate of grade 3 or 4 AEs with talimogene laherparepvec supports its use in combination with other therapies [6].

## Available data from combination studies of talimogene laherparepvec in melanoma

Based on the rationale for combining talimogene laherparepvec with agents that can promote T-cell responses, talimogene laherparepvec has been evaluated in clinical trials for melanoma in combination with ipilimumab or pembrolizumab. Data on combination therapy with intralesional therapies have, until recently, been lacking; therefore, these studies provide a benchmark for future research.

### Ipilimumab combined with talimogene laherparepvec

Ipilimumab is indicated in both Europe and the US for the treatment of advanced (unresectable or metastatic) melanoma in adults [32, 38] (see Camacho LH [39] for a recent review). It is a IgG1 monoclonal antibody directed against CTLA4, which blocks immunosuppression mediated by the interaction of B7 ligands (B7.1 and B7.2) on antigen-presenting cells and CTLA4 on CD8+ and CD4+ T cells and might deplete immunosuppressive regulatory T cells [36, 40, 41]. This disinhibits the expansion of T-cell responses, promoting the production of autoreactive T cells [36]. When administered as a monotherapy, ipilimumab demonstrated significant efficacy in patients with unresectable and metastatic melanoma, although immune-related AEs were common [42]. In a Phase III trial, ipilimumab was associated with a response rate of 10.9% and a median OS of 10.1 months [42]. OS data from a pooled analysis of ten prospective and two retrospective observational studies of ipilimumab, including two Phase III trials, indicate a 3-year survival rate of 22% with ipilimumab [43]. The survival rate plateaus around year 3 and is maintained up to 10 years in some patients [43]. Consequently, while efficacious, the low response rate and the frequency of severe AEs limit its use as a monotherapy.

The addition of ipilimumab to talimogene laherparepvec has the potential to enhance the priming and activation of T cells: dendritic cells present tumor antigens that are released following the oncolytic replication of talimogene laherparepvec (Fig. 2) [8, 36]. Talimogene laherparepvec was evaluated in combination with ipilimumab in the Phase Ib portion of an ongoing Phase Ib/II clinical trial (Study 20110264; clinicaltrials.gov identifier: NCT01740297). For this portion of the study, 21 patients were screened and 19 patients were enrolled across five US sites from February 2013 to July 2013 [44]. Patients with unresectable, injectable stage IIIB–IVM1c melanoma, who had received no prior systemic therapy (except prior adjuvant therapy ≥ 6 months from last therapy) were included in the study [44]. Talimogene laherparepvec was administered intralesionally as monotherapy at an initial dose of 10^6^ PFU/ml and then at 10^8^ PFU/ml every 2 weeks from week 4 [44]. Intravenous ipilimumab, administered at a dose of 3 mg/kg every 3 weeks for four infusions, began at the time of the third dose of talimogene laherparepvec [44]. Talimogene laherparepvec was continued until CR, all injectable tumors disappeared, progressive disease (PD) per modified immune-related response criteria (irRC), or drug intolerance [44]. The primary endpoint was the incidence of dose-limiting toxicities (DLTs), defined as any treatment-related non-laboratory grade ≥4 AE, grade ≥4 immune-mediated dermatitis or endocrinopathy, and grade ≥3 immune-mediated AE of any other type (e.g., pneumonitis, pancreatitis, nephritis, uveitis, and vasculitis).

No DLTs were observed during the DLT evaluation period or throughout the Phase Ib portion of the study [44]. Grade 3/4 treatment-related AEs were seen in 26% of patients (16% were attributed to talimogene laherparepvec and 21% were attributed to ipilimumab) (Table 2) [44]. ORR was 50% (95% CI, 26.0 to 74:0), which was almost double that observed in OPTiM (Table 2) [6, 42, 44]. This ORR was also higher than that observed in a Phase III trial with ipilimumab alone (data not shown), although it should be noted that patients were not required to have injectable disease in that Phase III study, so it is likely to have included a somewhat different patient population. Four patients (22%) had a confirmed CR [44], relative to 1.5% of patients treated with ipilimumab monotherapy in a Phase III trial [42]. All four patients were still in CR after a year [44]. Overall, 44% of patients in Study 20110264 had a DRR (defined as duration of response [DOR] lasting ≥6 months, where DOR is the interval from a first confirmed objective response to confirmed PD), compared with 16% of patients experiencing a DRR (defined as objective response lasting continuously ≥6 months) in OPTiM [6, 44]. Following the combination treatment, probabilities of 18-month PFS and OS were 50 and 67%, respectively [44].

**Table 2:** Key safety and efficacy data from the Phase 1b arm of clinical trial NCT01740297 (investigating ipilimumab in combination with talimogene laherparepvec in melanoma), the Phase 1b arm of clinical trial NCT02263508 (investigating pembrolizumab in combination with talimogene laherparepvec in melanoma), and historical data for talimogene laherparepvec monotherapy from the Phase III OPTiM clinical trial in melanoma*

	Talimogene laherparepvec + ipilimumab [44]	Talimogene laherparepvec + pembrolizumab [45]	Talimogene laherparepvec monotherapy [HISTORICAL DATA FROM OPTiM] [6, 30, 46]
*Baseline characteristics*			
Disease stage			
IIIB	1 (5)	1 (5)	22 (8)
IIIC	3 (16)	7 (33)	66 (22)
IVM1a	4 (21)	2 (10)	75 (25)
IVM1b	5 (26)	3 (14)	64 (22)
IVM1c	6 (32)	8 (38)	67 (23)
Unknown	0 (0)	0 (0)	1 (<1)
ECOG performance status			
0	14 (74)	19 (91)	209 (71)
1	5 (26)	2 (10)	82 (28)
Unknown	0 (0)	0 (0)	4 (1)
LDH			
≤ULN	15 (79)	16 (76)	266 (90)
>ULN	1 (5)	5 (24)	15 (5)
Unknown	3 (16)	0 (0)	14 (5)
*Safety findings*			
Grade 3/4 TRAE, *N* (%)			
Any event	5 (26)	7 (33)†	33 (11)
Any attributed to talimogene laherparepvec	3 (16)	4 (19)	33 (11)
Any attributed to checkpoint inhibitor	4 (21)	6 (29)†	NA
*Efficacy findings*			
ORR, *n* (%)	9 (50)	12 (57)	78 (26)
CR, *n* (%)	4 (22)	5 (24)	32 (11)
PR, *n* (%)	5 (28)	7 (33)	46 (16)
SD, *n* (%)	4 (22)	3 (14)	134 (45)
PD, *n* (%)	5 (28)	6 (29)	62 (21)
DRR, *n* (%)^‡^	8 (44)	NR	48 (16)
DCR, *N* (%)	NR	15 (71)	225 (76)
12-month PFS, %	50	71	NR
12-month OS, %	72	NR	74
Tumor response at the lesion level, %^#^			
Injected lesions	74	80	64
Non-injected lesions	52	35	NR
Non-visceral	54	45	34
Visceral	50	28	15

*CR* complete response, *DCR* disease control rate, *DRR* durable response rate, *ECOG* Eastern Cooperative Oncology Group, *LDH* lactate dehydrogenase, *NA* not applicable, NR not reported, *ORR* overall response rate, *OS* overall survival, *PD* progressive disease, *PFS* progression-free survival, *PR* partial response, *SD* stable disease, *TRAE* treatment-related adverse event, *ULN* upper limit of normal

*The data are derived from three independent clinical trials; comparisons across trials should be interpreted with caution. †Data are for grade 3 TREAs only; one grade 4 TRAE (pneumonitis, pembrolizumab related) was reported. ^‡^In of clinical trial NCT01740297, DRR is defined as a duration of response (DOR) lasting ≥6 months, where DOR is the interval from a first confirmed objective response to confirmed PD. In OPTiM, DRR was defined as an objective response lasting continuously ≥6 months. ^#^Tumor response defined as ≥50% regression

There was evidence of immune modulation during the study and total CD8 + T cells and activated CD8 + T cells were significantly increased from baseline following treatment with talimogene laherparepvec [44]. The increase in activated CD8+ T cells seemed to be greater in those patients experiencing disease control rather than progressive disease [44]. However, this differentiation was lost after ipilimumab administration [44]. CD4 + T cells expressing ICOS (inducible T-cell costimulator), an activation marker upregulated by CTLA-4 blockade, significantly increased from baseline at weeks 9 and 15 after ipilimumab was given, but not during the talimogene laherparepvec monotherapy period [44]. These immune findings indicate that T-cell responses with talimogene laherparepvec and ipilimumab may be complementary.

There are inherent limitations with comparing data across trials. For example, the distribution of patients across the disease stages differs between Study 20110264 and OPTiM and different assessment criteria were used. Nonetheless, these early phase findings suggest that the efficacy of combined ipilimumab and talimogene laherparepvec may be greater than that seen historically with ipilimumab or talimogene laherparepvec monotherapy [6, 42, 44].

### Pembrolizumab combined with talimogene laherparepvec

Pembrolizumab, recently reviewed by Khoja et al. [47], is indicated for the treatment of advanced (unresectable or metastatic) melanoma in adults (Europe and US) and for disease progression following ipilimumab and, if BRAF V600 mutant, a BRAF inhibitor (US) [33, 48]. Pembrolizumab is a monoclonal IgG4 antibody directed against PD-1, which blocks the immunosuppression mediated by the interaction of PD-L1 on tumor cells and PD-1 on CD8+ and CD4+ T cells, therefore, improving tumor cell recognition by T cells [36, 40, 49]. In the randomized Phase III KEYNOTE-006 trial (clinicaltrials.gov identifier: NCT01866319) that assessed the efficacy of pembrolizumab vs ipilimumab in advanced melanoma, pembrolizumab improved PFS, OS, and ORR and was associated with fewer grade ≥3 AEs [50].

The addition of pembrolizumab to talimogene laherparepvec has the potential to enhance the systemic antitumor response by enhancing the recognition and killing of tumor cells by T cells that have been primed as a result of talimogene laherparepvec injection (Fig. 2) [8, 36]. Talimogene laherparepvec was evaluated in combination with pembrolizumab in the Phase Ib portion of the Phase Ib/III clinical trial, called MASTERKEY-265 (clinicaltrials.gov identifier: NCT02263508) [45, 51]. For this portion of the study, 21 patients with stage IIIB–IVM1c melanoma with injectable lesions and no prior systemic therapy were enrolled from December 2014 to March 2015 at 11 institutions in Europe and the US [45, 51]. Talimogene laherparepvec was administered as monotherapy at an initial dose of 10^6^ PFU/ml and then at 10^8^ PFU/ml every 2 weeks from week 3 [45]. Pembrolizumab was administered intravenously at a dose of 200 mg every 2 weeks, commencing at the time of the third dose of talimogene laherparepvec [45]. Treatment with both therapies was continued for up to 2 years or until (whichever occurred first) CR or PD per modified irRC, intolerance, or, for talimogene laherparepvec only, when there are no longer any remaining injectable lesions [51]. The primary endpoint for the Phase Ib portion of the trial was the incidence of DLTs, with the evaluation period defined as 6 weeks from the initial administration of pembrolizumab [45, 51]. Data cutoff for the Phase Ib safety and efficacy results was January 4, 2016 [45]. The study met its primary endpoint with no DLTs observed during the monitoring period [45]. There was no additional toxicity with the combination treatment compared with that expected for the monotherapies (Table 2).

The combination therapy was associated with clinical benefit with a confirmed ORR of 57% and confirmed CR rate of 24% (Table 2) [45]. This ORR was greater than previously seen with pembrolizumab in a Phase III trial (34%) and with talimogene laherparepvec seen in OPTiM (26%)—although it should be recognized that cross-trial comparisons are associated with limitations, particularly as these studies had different designs and patient populations [6, 439]. Unconfirmed ORR was 67%, and unconfirmed CR rate was 29% [45]. Median PFS was not reached during the study, with 71% of patients being progression free at 6 months; disease control rate (DCR) was 71% [45]. As seen previously, an increase in circulating cytotoxic T cells (CD3+/CD8+) was observed after the start of talimogene laherparepvec monotherapy, as well as an upregulation of PD-1 and TIM-3 on these cells [45]. These results need careful interpretation, since melanoma patients with skin metastases often present with a less aggressive clinical course. However, the data still indicate that talimogene laherparepvec primes the immune response to enable an optimum response to pembrolizumab. Overall, these early findings suggest increased efficacy with combined pembrolizumab and talimogene laherparepvec treatment compared with pembrolizumab or talimogene laherparepvec monotherapy [6, 45, 50].

### Summary of available data from combination studies of talimogene laherparepvec in melanoma

Based on these early phase data, combining talimogene laherparepvec with ipilimumab or pembrolizumab may result in greater efficacy for patients than either therapy alone [6, 42, 44, 45, 50]. The ORR with talimogene laherparepvec plus either ipilimumab or pembrolizumab was around double that seen with talimogene laherparepvec alone, while the rate of CRs was more than doubled [6, 44, 45]. The combination clinical trials have not raised additional safety considerations, with safety profiles in line with those expected for either of these drugs as monotherapies.

## Ongoing talimogene laherparepvec combination studies in melanoma and other tumor types

### Melanoma

Based on the promising early results seen to date for talimogene laherparepvec combination therapy in melanoma, a number of clinical trials are ongoing (Table 3).

**Table 3:** Summary of ongoing clinical trials of talimogene laherparepvec in combination with immunotherapies

Trial name/registration number	Therapies	Design	Eligibility	Status and expected number of patients (*n*)	Estimated primary completion date
*Melanoma*					
Study 20110264 (NCT01740297; EudraCT 2012–000307–32)	Talimogene laherparepvec plus ipilimumab vs ipilimumab alone*	Randomized Phase II, multicenter, open-label trial	Adults with unresected, measurable stage IIIB–IVM1c melanoma with injectable cutaneous, subcutaneous, or nodal lesions will be included. Patients will be either treatment naïve or have received only one line of systemic anticancer therapy if BRAF wild-type or up to two lines of systemic anticancer therapy (including one BRAF inhibitor-containing regimen) if BRAF mutant	Ongoing *n* = 217	August 2016
MASTERKEY-265 (NCT02263508; EudraCT 2014–000185–22)	Talimogene laherparepvec plus pembrolizumab vs pembrolizumab plus placebo†	Randomized Phase III, multicenter, trial	Adults with unresected, measurable stage IIIB–IVM1c melanoma with injectable cutaneous, subcutaneous, or nodal lesions will be included. Patients with BRAFV600 wild-type tumors must not have received any prior systemic anticancer treatment. Patients with BRAFV600 mutated tumors may have received BRAF-targeted therapy. Patients must have a tumor sample that is adequate for PD-L1 assessment prior to randomization	Recruiting *n* = 660	May 2018
*Recurrent or metastatic SCCHN*					
MASTERKEY-232 (NCT02626000; EudraCT 2015-003011-38)	Talimogene laherparepvec plus pembrolizumab	Phase Ib/III multicenter, open-label trial	Adults with recurrent or metastatic SCCHN unsuitable for curative surgical resection or curative radiotherapy will be included. Disease must have progressed following treatment with a platinum-containing regimen and patients must be candidates for intralesional therapy administration	Recruiting *n* = 40 (Phase 1b) *n* = 62 (Phase III)	August 2019

*NR* not reported, *SCCHN* squamous cell carcinoma of the head and neck

*In the talimogene laherparepvec plus ipilimumab treatment arm, talimogene laherparepvec is being administered on day 1 of week 1, day 1 of week 4, then every 2 weeks thereafter, while ipilimumab is being administered on day 1 of weeks 6, 9, 12, and 15 (four infusions in total) [52]. In the ipilimumab only treatment arm, ipilimumab is being administered on day 1 of weeks 1, 4, 7, and 10 (four infusions in total) [52]

†In the talimogene laherparepvec plus pembrolizumab treatment arm, talimogene laherparepvec is being administered at day 1 of weeks 0, 3, 5, and 7 then every 3 weeks starting at day 1 of week 9, while pembrolizumab is being administered on day 1 of week 0, then every 3 weeks starting at day 1 of week 3 [51, 53]. In the pembrolizumab plus placebo treatment arm, pembrolizumab is being administered on day 1 week 0, then every 3 weeks starting at day 1 week 3 and placebo is being administered on day 1 of week 0, 3, 5, and 7 then every 3 weeks starting at day 1 week 9 [51, 53]

#### Ipilimumab plus talimogene laherparepvec

The Phase II portion of the Phase Ib/II clinical trial mentioned above (Study 210110264; clinicaltrials.gov identifier: NCT01740297), evaluating talimogene laherparepvec in combination with ipilimumab for patients with stage IIIB–IVM1c melanoma, includes a randomized design in which patients are receiving talimogene laherparepvec and ipilimumab or ipilimumab alone [41]. Approximately 200 patients across 40 sites in Europe and the US have been enrolled (enrolment is now complete) [52]. Patients are being treated with talimogene laherparepvec until CR, all injectable tumors have disappeared, disease progression per a modified irRC, or intolerance of study treatment [52]. The primary outcome is to evaluate efficacy as assessed by ORR [52]. Secondary outcomes include safety, best overall response, DCR, DRR, DOR, time to response (TTR), PFS, resection rate, OS, and landmark OS by year [52]. An interim analysis from this study was recently performed [54]. In the efficacy set, which consisted of 82 patients with ≥ 48 weeks of follow-up, the confirmed ORR for talimogene laherparepvec and ipilimumab was 36 versus 17.5% (ipilimumab alone), while the unconfirmed ORR was 50% (talimogene laherparepvec plus ipilimumab) versus 27.5% (ipilimumab) [54]. For the 165 patients in the Phase II safety set, no unexpected AEs were observed. The most common treatment-emergent AEs were chills, fatigue, pyrexia, pruritus, and rash. Grade 3/4 treatment-emergent AEs were similar between arms [54]. The data suggest that talimogene laherparepvec combined with ipilimumab has greater efficacy than either agent alone without additional safety concerns [54]. The primary analysis of response will occur 6 months after the last patient is randomized.

#### Pembrolizumab plus talimogene laherparepvec

The Phase III portion of the Phase Ib/III MASTERKEY-265 clinical trial (clinicaltrials.gov identifier: NCT02263508) is evaluating the safety and efficacy of talimogene laherparepvec in combination with pembrolizumab vs pembrolizumab plus intralesional placebo (talimogene laherparepvec formulation excipients) in patients with IIIB–IVM1c melanoma, and is currently enrolling patients [51]. Patients are being recruited across 21 sites in Europe, the US, and Australia [51]. It is expected that 660 patients will be randomized 1:1 to each treatment arm [51, 53]. Patients will be treated until 24 months from the date of the first dose of pembrolizumab or end of treatment due to disappearance of injectable lesions (talimogene laherparepvec/placebo only), confirmed CR (pembrolizumab discontinuation after confirmed CR is optional), disease progression per irRC-RECIST, or intolerance of study treatment [51]. Final analysis will occur 5 years after the last patient is enrolled in the Phase III portion of the study [53]. The primary outcome is to evaluate efficacy as assessed by PFS (centrally reviewed using modified RECIST 1.1) and OS and secondary outcomes include safety, ORR DCR, DRR, DOR, PFS (by modified irRC-RECIST), OS, and patient reported outcomes [51, 53].

### Other tumor types

Talimogene laherparepvec combination studies in additional cancers have been initiated. At present, there are ongoing studies investigating talimogene laherparepvec in combination with immunotherapies in solid tumors other than melanoma, including a Phase 1b/III multicenter, randomized, open-label trial (MASTERKEY-232 [clinicaltrials.gov identifier: NCT02626000]) in recurrent or metastatic squamous cell carcinoma of the head and neck. In the Phase Ib part of this trial, talimogene laherparepvec is being administered in combination with pembrolizumab to approximately 40 patients [55]. The primary endpoint will be DLT, which will be evaluated based on the first 18 DLT-evaluable patients [55]. An expansion cohort of an additional 22 treated patients will be enrolled to further evaluate the safety and to estimate the efficacy of the combination of talimogene laherparepvec with pembrolizumab to support a decision to initiate the Phase III study [55].

## Conclusions and future perspectives

Oncolytic immunotherapy is an active area of ongoing research with talimogene laherparepvec at the forefront of the field [1, 2]. Based on complementary mechanisms of action, clinical trials are in progress to extend the proven therapeutic benefit seen with talimogene laherparepvec monotherapy through combinations with other immunotherapies. The initial data from early studies in melanoma suggest that combining talimogene laherparepvec with ipilimumab or pembrolizumab is well tolerated and more efficacious than treatment with the individual therapies alone, with evidence that complementary mechanisms of action are responsible for the enhanced effects. The combination with pembrolizumab and talimogene laherparepvec is also being studied in other cancer types, such as SCCHN. Studies of solid tumors other than melanoma will help to ascertain whether talimogene laherparepvec has the capacity to engage the immune system in tumors that are currently not responsive to immunotherapy. Such tumors do not usually respond well checkpoint inhibitors, but if talimogene laherparepvec can effectively initiate an immune response that can be modulated by checkpoint inhibitors, this combination therapy may become an option for a larger patient population.

There is also potential for future combination trials of talimogene laherparepvec with other immunotherapies that are currently in development. For example, agents that target immunosuppressive tumor-associate macrophages [56], immune-activating agents, such as cytokines [57] and STING (Stimulator of Interferon Genes)-activating agents [58], and agents that target T-cell costimulatory receptors molecules [59] could potentially be investigated in combination with talimogene laherparepvec in the future. Furthermore, radiation therapy or chemotherapy may also prove to be effective partners for talimogene laherparepvec or talimogene laherparepvec combination regimens [60–62]. In addition to direct tumor cell cytotoxicity, localized radiation therapy and chemotherapeutic agents can also lead to systemic responses through immunomodulatory effects both on the tumor and the microenvironment [61, 63, 64]. This process can prime tumors for an immune-mediated response and may enhance efficacy as part of a combination strategy. Indeed, combining radiation therapy with checkpoint inhibitors has demonstrated promising results [63, 65], as have combinations of oncolytic virotherapies with chemotherapies [61, 64], and trials of talimogene laherparepvec combined with radiotherapy or chemotherapy are being initiated [66–69]. Finally, increased durable disease control might also be achieved by combining talimogene laherparepvec with targeted therapies, such as BRAF inhibitors and vascular endothelial growth factor inhibitors, and this could be useful in situations where long-term effectiveness can otherwise be limited by the emergence of resistance [70].

In the future, precision/personalized therapy may be achieved via talimogene laherparepvec combination therapy by tailoring the choice of combination agent to the individual patient and tumor characteristics, and studies are underway to identify potential predictive biomarkers. Further clinical research will help establish the full potential for talimogene laherparepvec in combination with other agents for the treatment of cancer.

## Abbreviations

AE: Adverse events
CR: Complete response
DCR: Disease control rate
DLT: Dose-limiting toxicity
DOR: Duration of response
DRR: Durable response rate
EAC: Endpoint-assessment committee
FDA: Food and Drug Administration
GM-CSF: Granulocyte–macrophage colony-stimulating factor
HR: Hazard ratio
HSV-1: Herpes simplex virus type 1
ICOS: Inducible T-cell costimulator
ORR: Overall response rate
PD: Progressive disease
PFS: Progression-free survival
PKR: Protein kinase R
PR: Partial response
SCCHN: Squamous cell carcinoma of the head and neck
STING: Stimulator of interferon genes
TTR: Time to response
